# Supplementary material for: Profiles of Free and Bound Phenolics and Their Antioxidant Capacity in Rice Bean (Vigna umbellata)
Source: Foods. 2023 Jul 16;12(14):2718. doi: 10.3390/foods12142718 (PMC10378860; doi:10.3390/foods12142718)
Supplement: Supplementary file 1 [file foods-12-02718-s001.zip › foods-2431733-supplementary.pdf]

Supplementary material

Identification of individual phenolic compounds in various extracts

The compound 1 showed the parent ion [M-H]- at *m/z* 191.056, and corresponded to fragment ions at *m/z* 127.039, which was identified as quinic acid. Phloroglucinol (peak 2), showed the parent ion [M-H]- at *m/z* 127.039, and fragment ion *m/z* 109.029 [(M+H)-H_2O]- upon loss of one molecule of water. Due to the parent ion of compound 3 at *m/z* 169.013 [M-H]- and the fragment at *m/z* 125.023 [M-H-CO_2]-, the compound was positively identified as gallic acid. With a deprotonated molecular ion at *m/z* 305.0677 [M-H]- and fragments at *m/z* 261.077 [(M-H)-C_2H_4O]- and 179.034 [(M-H)-$C_6H_6O_3$]-, compound 4 was tentatively recognized as gallocatechin. According to the deprotonated molecular ion, *m/z* 153.018 (M-H), compound 5 was tentatively identified as protocatechuic acid, which gave rise to secondary fragment ion *m/z* at 109.028 by loss of carboxyl group. Compound 6 was identified as 3,4-dihydroxyphenylacetic acid by the deprotonated molecular ion *m/z* 167.034 (M-H) and the product ions *m/z* 123.044 as measured. Compound 7 and compound 10 showed the parent ions at *m/z* 577.135 (M-H) and their MS/MS spectra exhibited the same fragmentation pattern, and fragment ions were *m/z* 451.103 [M-H-H_2O-ring B]-, 407.077 [(M-H)-170Da]-. Compounds 7 and 10 were positively identified as procyanidin B1 and B2, respectively, by comparing their retention times to standard references. Compounds 8 and 14 were identified as catechin and epicatechin due to their characterized deprotonated molecular ion *m/z* 289.072 (M-H) and their fragment ion at *m/z* 245.081 [M-H-$(CH)_2OH$]- and 179.034 [M-H-B-ring]-, and also their retention time comparable to these two standard compounds. Peak 9 was definitively identified as *p*-hydroxybenzoic acid after the deprotonated molecular ion at *m/z* 137.023 produced the fragment ion at *m/z* 93.033 by loss of a carboxyl group (-44 Da). Component 11 was tentatively confirmed as catechol by its parent ion [M-H]- at *m/z* 109.028 and its fragmentation ion at *m/z* 91.018 due to a lost methyl group. Compound 12 exhibited parent ion at *m/z* 179.034 [M-H]-, which yield fragment ions at *m/z* 135.044 due to loss of carboxyl moiety (-44 Da). Therefore, compound 12 was identified as caffeic acid. Compound 13 was characterized with the deprotonated molecular ions *m/z* 197.046 (M-H) and product ions *m/z* at 182.022 and 166.998, which was confirmed to be syringic acid referring to earlier research [34]. According to the similar fragmentation pattern reported by Chen et al. [35], compound 16 displayed a parent ion at *m/z* 609.146 (M-H), the corresponding product ions *m/z* 300.028 was formed by the loss of rutinoside moiety (309 Da) from the C-3 position of the quercetin structure in the parent, which usually occurs when rutin cleaved to form quercetin. Thus, compound 16 was identified as rutin. Compound 17 and 19 showed the parent ions at *m/z* 300.999 (M-H) and at *m/z* 163.039 (M-H), respectively, and produced the fragment [M-H-44 Da]- ions at *m/z* 257.009 and *m/z* 119.049 due to the loss of carboxyl group, and thus were tentatively identified as ellagic acid and *p*-coumaric acid. The parent ions of compounds 18 and 20 were both *m/z* 431.098 (M-H) and had indistinguishable precursor fragmentation patterns, with product ions *m/z* 311.056 [M-H-$C_4H_8O_4$]- and *m/z* 341.066 [M-H-$C_3H_6O_3$]-. However, the most abundant product ion of vitexin was *m/z* 311.056 [M-H-$C_4H_8O_4$]-, which was different from that of isovitexin with the main product ion at 341.066 [M-H-$C_3H_6O_3$]-. Therefore, Compounds 18 and 20 were positively identified as isovitexin and vitexin, respectively [36]. Compound 21 was preliminarily identified as isoquercitrin based on the deprotonated molecular ion at *m/z* 463.088 (M-H), and its fragment ions at *m/z* 300.027 and *m/z* 301.035. Compound 22 was likely to be sinapic acid since it had a deprotonated molecular ion of *m/z* 223.061 (M-H), fragment ions of *m/z* 193.013 [M-H-CH_2O]-, and a primary product ion *m/z* 208.037 after the loss of the methyl group. Compound 23 was formally identified as ferulic acid due to that it displayed deprotonated molecular [M-H]- ion at *m/z* 193.050, and produced a fragment ion at *m/z* 178.026 [M-H-CH3]- after losing a molecule of methyl group in the negative ion mode, which in turn continues to lose a molecule of CO_2 to form a fragment ion *m/z* 149.060 [M-H-CH_3-CO_2]-. Compound 24's

deprotonated ion, *m/z* 431.099 (M-H), was used to make a preliminary as genistin, and fragment ions [M-H-$C_6H_{10}O_5$]- and [M-H-glucose-OH]- were produced at *m/z* 269.045 and *m/z* 268.038. According to the previous report by Alakolanga et al. [37], compound 25 exhibited a parent (M-H)- ion at *m/z* 593.152, and produced a fragment ion at *m/z* 285.040 [M-H-308Da]- with kaempferol characteristic by loss of rutin glycosides (including 146 Da rhamnoside and 162 Da glucoside moieties). Compound 25 was identified as kaempferol-3-0-rutinosided. Compound 28 was most likely to be morin with deprotonated ion 301.036 (M-H), and generated fragment ions with respective mass values of *m/z* 273.040 and at *m/z* 255.030. Compared with the reported literature [38], compound 29 was tentatively identified as daidzein, which are based on the product ions *m/z* 135.009 [M-H-C_8H_6O]- and *m/z* 107.133 [M-H-$C_6H_{11}O_5$]- produced by the parent ion *m/z* 253.051 (M-H). Compound 30 produced the fragment ions at *m/z* 268.037 by loss of a CH_3 (methyl group), and the parent ions at *m/z* 283.061 (M-H). Therefore, it was formally identified as glycitein. Compound 31 was tentatively assigned as quercetin according to parent [M-H]- ion at *m/z* 301.035, and fragment ion at *m/z* 178.998 due to B ring cleavage following the RDA (retro Diels-Alder) ring opening mechanism, which further lost one carbonyl group to form the fragmentation ion at *m/z* 151.003 [39]. Compound 32 ([M-H]- at *m/z* 147.044) produced product ions at *m/z* 102.947 after losing one carboxyl group, which was recognized as trans-cinnamic acid. Compound 33 displayed parent ions at *m/z* 269.046 (M-H) and MS/MS fragment ions at *m/z* 133.028 (by loss of $C_8H_8O_2$) which was consistent with the genistein's MS data [40], thus, genistein was a likely identification for compound 33. Naringenin was identified as compound 34, with deprotonated molecular ion [M-H]- at *m/z* 271.061, and showed the fragmentation ions at *m/z* 151.003 [$A_{1,3}$-H] and *m/z* 119.049 [M-H-$C_7H_4O_4$]-. The deprotonated molecular ion of compound 35 was *m/z* 285.041 and product ion at *m/z* 257.046 (by loss of one molecular CO). It was confirmed as kaempferol. In addition to the above identified compounds, compounds 15, 26 and 27 were identified as daidzin, taxifolin and benzoic acid through systematic comparison of the parent ion, fragmentation ion and retention time of standard compounds.

References

34. Barnaba, C.; Nardin, T.; Pierotti, A.; Malacarne, M.; Larcher, R., Targeted and untargeted characterisation of free and glycosylated simple phenols in cocoa beans using high resolution-tandem mass spectrometry (Q-Orbitrap). *J. Chromatogr. A* **2017,** *1480*, 41-49.

35. Chen, Y. H.; Zhang, H.; Liu, R. H.; Mats, L. L.; Zhu, H. H.; Pauls, K. P.; Deng, Z. Y.; Tsao, R., Antioxidant and anti-inflammatory polyphenols and peptides of common bean (*Phaseolus vulga* L.) milk and yogurt in Caco-2 and HT-29 cell models. *J. Funct. Food.* **2019,** *53*, 125-135.

36. Gai, Q. Y.; Jiao, J.; Wang, X.; Fu, Y. J.; Lu, Y.; Liu, J.; Wang, Z. Y.; Xu, X. J., Establishment of *Cajanus cajan* (Linn.) Millsp. cell suspension cultures as an effective in vitro platform for the production of pharmacologically active phenolic compounds. *Ind. Crop. Prod.* **2020,** *158*, 11.

37. Alakolanga, A.; Siriwardane, A.; Kumar, N. S.; Jayasinghe, L.; Jaiswal, R.; Kuhnert, N., LC-MSn identification and characterization of the phenolic compounds from the fruits of *Flacourtia indica* (Burm. F.) Merr. and *Flacourtia inermis* Roxb. *Food Res. Int.* **2014,** *62*, 388-396.

38. Mecha, E.; Leitao, S. T.; Carbas, B.; Serra, A. T.; Moreira, P. M.; Veloso, M. M.; Gomes, R.; Figueira, M. E.; Brites, C.; Patto, M. C. V.; Bronze, M. R., Characterization of soaking process' impact in common beans phenolic composition: Contribute from the unexplored portuguese germplasm. *Foods* **2019,** *8* (8), 27.

39. da Costa, M. F.; Galaverna, R. S.; Pudenzi, M. A.; Ruiz, A.; de Carvalho, J. E.; Eberlin, M. N.; dos Santos, C., Profiles of phenolic compounds by FT-ICR MS and antioxidative and antiproliferative activities of *Stryphnodendron obovatum* Benth leaf extracts. *Anal. Methods* **2016,** *8* (31), 6056-6063.

40. John, K. M. M.; Harnly, J.; Luthria, D., Influence of direct and sequential extraction methodology on metabolic profiling. *J. Chromatogr. B* **2018,** *1073*, 34-42.
